# Qualitative study of experience of acceptance and commitment therapy (ACT+) amongst Survivors' Rehabilitation Evaluation after Cancer (SURECAN) trial participants and therapists: A protocol.

**DOI:** 10.3310/nihropenres.13382.1

**Published:** 2023-05-12

**Authors:** Sheila Donovan, Trudie Chalder, Dipesh Gopal, Imran Khan, Ania Korszun, Elisavet Moschopoulou, Damien Ridge, Clare Robinson, Stephanie Taylor, SURECAN Investigators

**Affiliations:** 1 Wolfson Institute of Population Health, Queen Mary University of London, London, UK; 2 Department of Psychological Medicine, Kings College London, London, UK; 3 School of Social Sciences, University of Westminster, London, UK

**Keywords:** Cancer, Quality of Life, Qualitative, Protocol, Acceptance and Commitment Therapy

## Abstract

**Background:**

This interview study forms part of a mixed methods process evaluation of the Survivors’ Rehabilitation Evaluation after Cancer (SURECAN) trial to understand the experiences of participants (who are living with and beyond cancer) in receiving a form of acceptance and commitment therapy, and therapists providing the intervention. SURECAN is a multi-centre, pragmatic, individual participant randomised controlled trial of an intervention based on acceptance and commitment therapy supplemented by support for return to meaningful work and/or physical activity (ACT+). This qualitative study addresses the ways in which participants believe they benefit from ACT+ (or not), and how the ACT+ intervention might best be implemented into routine National Health Service (NHS) care.

**Methods:**

The study investigates experiences of ACT+ by different participants to understand how we can optimise the ACT+ intervention and its delivery (assuming the intervention is successful). We will conduct individual interviews with participants who have taken part in the active arm of the SURECAN trial to understand their experiences of engaging with and receiving ACT+, their perceptions of the impact of the therapy, and relevant contextual factors influencing these experiences. In particular, we will focus on comparing our interview findings between those trial participants who improved and those who failed to improve (or worsened), in terms of quality of life following ACT+. Additionally, we will conduct individual interviews with therapists who have delivered ACT+ as part of the SURECAN trial, to understand their experiences of delivering ACT+.

**Conclusions:**

Consistent with other qualitative protocols, this protocol is not registered. Instead, it is shared as a means of documenting ahead of time, how we are endeavouring to understand the ways in which a newly trialled talking therapy is received by patients and therapists, and how (if successful) it might be incorporated into the NHS.

## Plain English summary

Our study looks at experiences of the SURECAN trial for people who are living with and beyond cancer. SURECAN is a trial of a kind of talking therapy called 'acceptance and commitment therapy'. Acceptance and commitment therapy (ACT) helps people to embrace their feelings and thoughts (and live according to their values) rather than fighting against them. Our therapists also talk to patients about their work life and levels of physical activity to help them get a balance (so we call it ACT+). Our study will interview participants (patients and therapists) on the trial about the benefits of ACT+ (or not), and how ACT+ therapy could become part of everyday NHS care. The study aims to work out how we can optimise the ACT+ therapy for the NHS. We will conduct individual interviews with participants who have received ACT+ in the SURECAN trial, to understand their experiences of ACT+, their perceptions of the talking therapy, and whether anything else going on in their lives at the time could explain their experiences of ACT+. In particular, we will compare trial participants whose quality of life improved with those who did not improve (or worsened). Additionally, we will talk with the ACT+ talking therapists to understand their experiences of delivering ACT+ to people who are living with and beyond cancer.

## Introduction

This interview study will form part of the mixed methods process evaluation of the Survivors’ Rehabilitation Evaluation after Cancer (SURECAN) trial, which will be conducted following Medical Research Council guidance ^1^. SURECAN is a multi-centre, pragmatic, individual participant randomised controlled trial of an intervention based on acceptance and commitment therapy (a talking therapy) supplemented by support for return to meaningful work and/or physical activity, according to the preferences of the individual study participant, known as ‘ACT+’. The ACT+ intervention in addition to usual aftercare is compared to usual aftercare only, for patients living with and beyond cancer (SURECAN Trial IRAS: 260823 Protocol v3.0 06/02/2022). Trial participants comprise individuals who have completed treatment with curative intent for one of five cancer groups (breast, lower gastrointestinal, haematological, head and neck, urological) and are experiencing low quality of life as assessed by the Functional Assessment of Cancer Therapy: General scale (FACT-G) ^2^. Trial participants are recruited through participating hospital cancer clinics, and the ACT+ intervention is delivered by trained therapists working in either participating IAPT services in primary care mental health services or the charity sector.

## Purpose

This interview study addresses the ways in which participants believe they benefit from ACT+ (or not), and how the ACT+ intervention might best be implemented into routine National Health Service (NHS) care. The purpose of the study is to investigate the experience of ACT+ by different participants to understand how we can optimise the ACT+ intervention and its delivery (assuming the intervention is successful). In particular, we will focus on comparing our interview findings from those trial participants who improved and from those who failed to improve (or worsened), in terms of quality of life following ACT+. We will also capture the experience of therapists who delivered the ACT+ intervention.

We will investigate experiences of ACT+, and ACT+ delivery, in two parts:

In Part A we will conduct individual interviews with participants who have taken part in the active arm of the SURECAN trial to understand their experiences of engaging with and receiving ACT+, their perceptions of the impact of the therapy, and relevant contextual factors influencing these experiences.

In Part B we will conduct individual interviews with therapists who have delivered ACT+ as part of the SURECAN trial, to understand their experiences of delivering ACT+ to people who are living with and beyond cancer.

## Theoretical framework

We will draw on Normalisation Process Theory ^3^, a theory that focuses on how innovations are incorporated into systems like the NHS. This approach essentially means that in our lines of questioning both participants and therapists, we will ensure to cover specific contexts of the trial; coherence (i.e. how people make sense) of the approaches used; cognitive participation (how people think about the delivery of the innovation); collective action (what people do to deliver an innovation); and reflective monitoring (how people evaluate their contributions and/or the consequences of the trial). This will ensure we ask pertinent questions of both trial participants and therapists; that we elicit narratives in order to explore how trial participants subjectively appraise their experiences related to ACT+; and explore how to best integrate ACT+ into the NHS should the therapy prove useful ^4, 5^.

## Research questions

Our research questions for part A are:

1) What are the differences in treatment perceptions and experiences between those trial participants who improved and those who did not following ACT+?
2) Why might different kinds of participants do better than others with ACT+?
3) How do participants explain the influence of life contexts on their outcomes?
4) How can we optimise the ACT+ intervention and its delivery, with regard to future implementation?

Our research question for part B is:

1) How can we optimise the ACT+ intervention and its delivery, with regard to future implementation?

## Sample and recruitment for part A

### Eligibility criteria

The inclusion criteria are:

1. participant in intervention arm of trial
2. received at least four sessions of ACT+
3. no longer receiving ACT+

The exclusion criteria are:

1. did not give consent to be approached for an interview
2. more than 14 months since final ACT+ session

### Sampling

***Size of sample.*** We aim to recruit up to 30 participants randomised to the intervention arm of the trial.

***Sampling strategy.*** We will conduct purposive sampling to obtain variation in participant characteristics. Dimensions of interest are cancer group, age, gender, and ethnic group (White, Black or Black British, Asian or Asian British, Mixed, Other), although other dimensions of interest may emerge iteratively.

### Recruitment

***Sample identification.*** A list of participants eligible for this study, and their demographic characteristics, will be extracted from the SURECAN trial database. Data extraction will take place while the trial is live.

From this list of eligible participants a sample of participants will be selected to approach for interview. This sample will be selected to provide variation in participant characteristics like cancer group, age, gender, and ethnicity. Where multiple participants share the same characteristics the selections from that group will be made randomly. Once participants have been approached for interview they will be removed from any future eligible participant lists.

The process of sample selection will be iterative, with the first sample chosen to provide overall diversity but assigning more weight to selecting a variety of different ‘cancer groups’ as far as possible, the aim being to identify a group of potential participants who have been treated for different cancers. We will not aim to identify equal numbers for each cancer group as it is likely that not everyone invited into this interview study will agree to participate. The trial statistician (CR) will work closely with the qualitative researcher (SD) to determine how many trial participants need to be identified in each sampling cycle.

When interviews have been conducted with individuals recruited from the first sample selected, information regarding their cancer group, age, gender, and ethnicity (available from the extracted data and confirmed with participants at the time of interview) will be collated by the qualitative researcher to produce an overview of the variation in the sample to date. This information on the make-up of the sample will be reviewed by the research team to determine which of the categories (our dimensions of interest) should receive more weight in the second sample selection in order to increase the variation in the sample. The need for any subsequent sample selection will be determined in a similar way. The need for any subsequent data extraction/s will depend on the number of participants recruited for interview from the samples selected (as described above) in relation to our target sample size of up to 30 interviewees.

**Figure 1.**
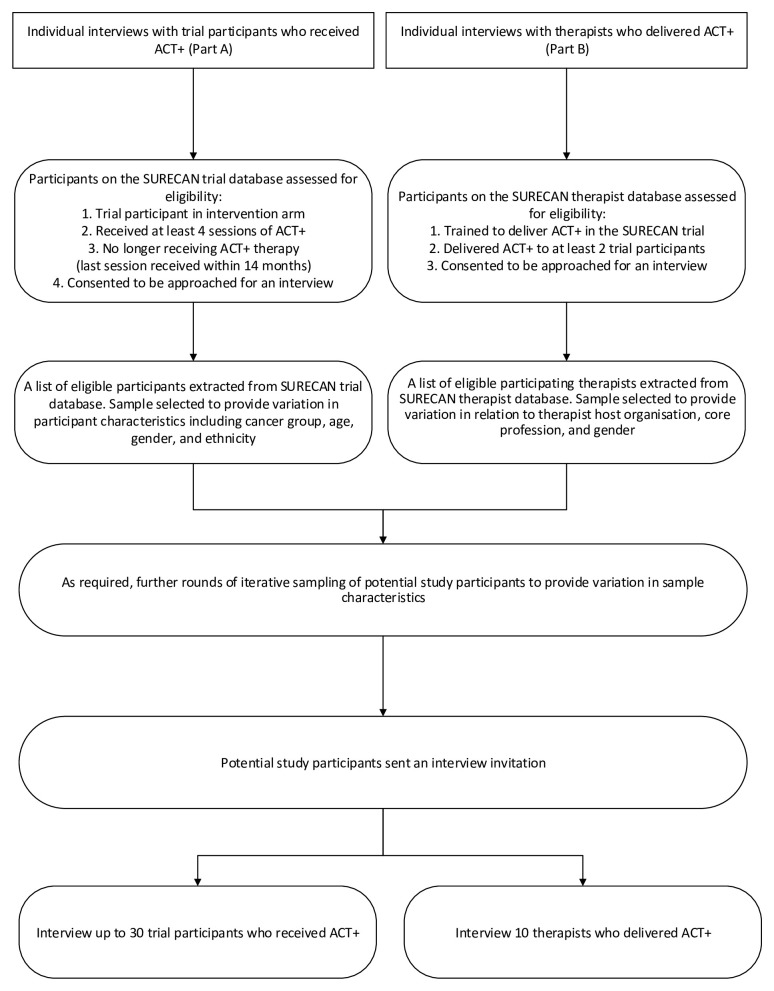
Study flow diagram.

The qualitative researcher will liaise with the interview study lead (DR), the trial manager (IK), the trial statistician (CR), and the research team at regular intervals to review how the process of forming the sample is progressing and to agree the timing and objectives of any subsequent data extraction/s. See Figure 1 (Study flow diagram) for an illustration of how participants for Part A will be identified.

***Consent.*** Consent to be approached about post-therapy interviews was sought at the time that consent to participate in the trial was obtained.

Initial contact will be made by post or email. The qualitative researcher will send potential participants, by post or electronically, an invitation pack containing an invitation letter, study information sheet, consent (or e-consent) form, and prepaid envelope (where appropriate) to return the consent form. The invitation letter will explain that the researcher can be contacted for further information and to address any queries. Between seven and 10 days after posting the invitation pack (and if the consent form has not been returned), the qualitative researcher will follow up with a telephone call to discuss the individual’s potential participation and answer any questions they have about the study. Subsequent to the invitation letter, a total of up to three phone attempts, and one email attempt (if appropriate) will be made to speak/communicate with the potential participant over a 30-day period. No further attempt will be made to make contact.

The researcher will explain to potential participants that although invited to participate in an interview, their involvement is entirely voluntary, and they can stop the interview at any time, no questions asked.

## Sample and recruitment for part B

### Eligibility criteria

The inclusion criteria are:

1. therapist trained to deliver ACT+ in the SURECAN trial
2. delivered ACT+ sessions to at least two trial participants

The exclusion criterion is:

1. did not give consent to be approached for an interview

### Sampling

***Size of sample.*** We aim to recruit 10 therapists participating in the SURECAN trial.

***Sampling strategy.*** We will conduct purposive sampling so as to include a range of experiences and views. Dimensions of interest for the sampling of therapist participants are therapist’s host organisation, core profession, and gender.

### Recruitment

***Sample identification.*** A list of therapists eligible for this study, and details of their host organisation, core profession, and gender, will be extracted from the SURECAN therapist database. From this list, a purposive sample selected to provide variation in the dimensions of interest will be approached for interview. See Figure 1 (Study flow diagram) for an illustration of how participants for Part B will be identified.

***Consent.*** Consent to be approached about post-intervention delivery interviews was sought at the time that consent to participate in the trial was obtained.

Initial contact will be made by email. The qualitative researcher will send an invitation pack (containing an invitation letter, study information sheet, and e-consent form) to potential participants electronically. The invitation letter will explain that the researcher can be contacted for further information and to address any queries. Between seven and 10 days after sending the invitation pack, (and if the consent form has not been returned), the qualitative researcher will follow up with an email, to remind the therapist about the invitation pack, ask if they have any questions about the study, and offer to speak on the phone at a convenient time to discuss their possible participation. Subsequent to the invitation letter, up to five reminders via email and/or phone will be made during a period of 30 days. No further attempt will be made to make contact.

The researcher will explain to potential participants that although invited to participate in an interview, their involvement is entirely voluntary, and they can stop the interview at any time, no questions asked.

## Participant involvement

Participants in this interview study (Parts A and B) will take part in a one-off, individual semi-structured interview, conducted either by telephone or via a data protection-compliant online platform (Skype or Microsoft Teams), whichever is their preference. Interviews will last for 40 to 60 minutes.

## Data collection

The use of a semi-structured interview approach ^6^ will i) allow us to address the same topics in each set of interviews and in so doing, generate comparable data about participants’ experience of receiving or delivering the ACT+ intervention, and ii) provide sufficient flexibility within the interviews to enable participants to highlight their concerns and elaborate on particular aspects of their accounts.

Topics for interviews with trial participants (Part A) will include the decision to take part in the SURECAN trial, expectations of the therapy, concerns about the therapy, understanding of ACT+, barriers and facilitators to ACT+, engagement in the ACT+ sessions, use of the ACT+ Participant Handbook, perceived impact of the therapy, why ACT+ worked/did not work, anything important going on at the time of ACT+, challenges emerging after completing the course of therapy.

Topics for interviews with therapists (Part B) will include working with the client group (people living with and beyond cancer), delivering the therapy in a trial context, delivery of ACT+ sessions, use of the ACT+ Therapist Manual, ending the therapy, perceived value of ACT+ for the client (their allocated trial participant).

## Data analysis and data management

### Data analysis

Interviews will be audio-recorded and transcribed *verbatim* by a professional transcribing service with which the university has an agreement, including to treat audio recordings and the resultant transcripts as strictly confidential. The qualitative researcher will review transcripts against the audio recordings to correct any errors and remove any identifying information.

Data will be managed in the qualitative data analysis software environment NVivo. All transcripts, once checked for accuracy and anonymised, will be uploaded to NVivo and coded. A close thematic analysis of the data will be conducted to identify ‘repeated patterns of meaning’ ^7^. The analysis will incorporate a ‘constant comparison’ approach, to ensure that relevant data are compared with similar data systematically ^8^.

***Blinding.*** Initially, analysis of the trial participant interview data set will be conducted using baseline data only. When the SURECAN trial has been completed and we are unblinded to the study outcomes, we will conduct further analysis, comparing interview findings from participants who improved and those who did not improve following ACT+.

The data extraction to identify eligible participants will be conducted by a statistician independent to the SURECAN trial to ensure the SURECAN trial statisticians remain blind to treatment group allocation of participants.

### Data management

Information related to participants will be kept confidential and managed in accordance with the General Data Protection Regulation (GDPR), NHS Caldicott Principles, The Research Governance Framework for Health and Social Care, and the conditions of Research Ethics Committee Approval.

The study information sheet will set out arrangements relating to confidentiality, security, storage and accessibility of data only to the study team.

The signed consent forms will kept in a locked cabinet at Queen Mary, University of London, accessible by authorised study staff only. All data collected will be fully anonymised by a unique participant ID. For telephone interviews, the qualitative researcher will use an encrypted digital audio-recorder to record the interview. The recording will be downloaded onto a secure and encrypted USB storage device immediately following the interview. For interviews conducted using a secure online calling platform, the recording function of the secure platform will be used to record the interview. The recording will be downloaded onto a secure and encrypted USB storage device immediately following the interview. Encrypted USBs are kept in a locked cabinet in a locked room.

A copy of the recordings will be downloaded onto an encrypted USB storage device and sent securely to a professional transcriber for transcription. The transcriber will upload the transcribed documents onto the USB storage device and return it securely to the study team.

All recording file data will be uploaded onto a dedicated folder on the secure virtualised environment at the Barts Cancer Centre (BCC) at Queen Mary, University of London, and deleted from the digital recorder and, after analysis, the encrypted storage devices. The folders where the data are stored will be accessible only to the appropriate members of the SURECAN study team.

## Ethical and regulatory considerations

### Research ethics approval

A favourable opinion from a Health Research Authority Research Ethics Service for the study protocol, consent forms, invitation letters and participant information sheets has been obtained ( **IRAS Number** 314406, **REC Number** 22/SW/0157).

### Ethical considerations

The Co-Chief Investigators will ensure that the study is carried out in accordance with the ethical principles in the Research Governance Framework for Health and Social Care, Second Edition, 2005, and its subsequent amendments as applicable together with applicable legal and regulatory requirements.

The informed consent process has been described in the consent section above. Consent materials comprise a study information sheet, an invite letter, and a consent form. We have made a particular effort to use clear, accessible language in these documents and have received advice on them from our study patient advisors. The information sheet covers the purpose of the study, why potential participants have been approached to take part and what would it mean for them if they chose to participate, the benefits and risks of participation, assurance that participation is voluntary and that withdrawal from the study can be at any time, the type of data collection, data storage, confidentiality and security, who the study is funded and sponsored by, who reviewed the study, and whom to contact for further information. Participants will be given a copy of their signed consent form at the time of their recruitment into the study.

There is potential for patient participants to become upset about their situation or their condition. If an interviewee becomes distressed, the interviewer will stop the interview and will stay with the participant while they recover, and check in with such participants by telephone in the days subsequent to the interview. Information as to how they can seek further help will be offered to participants.

## Sponsorship and indemnity

Queen Mary University of London will be the study sponsor. The sponsorship will be given on the basis of meeting the ‘Conditions of sponsorship’ which means that the research should be conducted and managed as per the Research Governance Framework for Health and Social Care 2005 and/or the Medicines for Human Use (Clinical Trials) Regulations 2004.

Queen Mary University of London has a no-fault indemnity insurance policy for research participants. These compensation arrangements apply where harm is caused to a participant that would not have occurred if they had not taken part in the study. These arrangements do not affect participants’ rights to pursue a claim through legal action.

## Data Availability

No data are associated with this article.

## References

[1] Moore GF Audrey S Barker M : Process evaluation of complex interventions: Medical Research Council guidance. *BMJ.* 2015;350:h1258. 10.1136/bmj.h1258 25791983 PMC4366184

[2] Cella DF Tulsky DS Gray G : The Functional Assessment of Cancer Therapy scale: development and validation of the general measure. *J Clin Oncol.* 1993;11(3):570–9. 10.1200/JCO.1993.11.3.570 8445433

[3] Murray E Treweek S Pope C : Normalisation process theory: a framework for developing, evaluating and implementing complex interventions. *BMC Med.* 2010;8:63. 10.1186/1741-7015-8-63 20961442 PMC2978112

[4] Greenhalgh T Hurwitz B : Why study narrative? *BMJ.* 1999;318(7175):48–50. 10.1136/bmj.318.7175.48 9872892 PMC1114541

[5] Hurwitz B Greenhalgh T Skultans V (Eds.) : Narrative research in health and illness.John Wiley & Sons.2008. Reference Source

[6] McIntosh MJ Morse JM : Situating and constructing diversity in semi-structured interviews. *Glob Qual Nurs Res.* 2015;2:2333393615597674. 10.1177/2333393615597674 28462313 PMC5342650

[7] Braun V Clarke V : Using thematic analysis in psychology. *Qual Res Psychol.* 2006;3(2):77–101. 10.1191/1478088706qp063oa

[8] Glaser BG : The constant comparative method of qualitative analysis. *Social Problems.* 1965;12(4):436–445. 10.2307/798843

