# Qualitative study of experience of acceptance and commitment therapy (ACT+) amongst Survivors' Rehabilitation Evaluation after Cancer (SURECAN) trial participants and therapists: A protocol.

**DOI:** 10.3310/nihropenres.13382.2

**Published:** 2024-01-03

**Authors:** Sheila Donovan, Trudie Chalder, Dipesh Gopal, Imran Khan, Ania Korszun, Elisavet Moschopoulou, Damien Ridge, Clare Robinson, Stephanie Taylor, SURECAN Investigators

**Affiliations:** 1 Wolfson Institute of Population Health, Queen Mary University of London, London, UK; 2 Department of Psychological Medicine, Kings College London, London, UK; 3 School of Social Sciences, University of Westminster, London, UK

**Keywords:** Cancer, Quality of Life, Qualitative, Protocol, Acceptance and Commitment Therapy

## Abstract

**Background:**

This interview study forms part of a mixed methods process evaluation of the Survivors’ Rehabilitation Evaluation after Cancer (SURECAN) trial to understand the experiences of participants (who are living with and beyond cancer) in receiving a form of acceptance and commitment therapy, and therapists providing the intervention. SURECAN is a multi-centre, pragmatic, individual participant randomised controlled trial of an intervention based on acceptance and commitment therapy supplemented by support for return to meaningful work and/or physical activity (ACT+). This qualitative study addresses the ways in which participants believe they benefit from ACT+ (or not), and how the ACT+ intervention might best be implemented into routine National Health Service (NHS) care.

**Methods:**

The study investigates experiences of ACT+ by different participants to understand how we can optimise the ACT+ intervention and its delivery (assuming the intervention is successful). We will conduct individual interviews with participants who have taken part in the active arm of the SURECAN trial to understand their experiences of engaging with and receiving ACT+, their perceptions of the impact of the therapy, and relevant contextual factors influencing these experiences. In particular, we will focus on comparing our interview findings between those trial participants who improved and those who failed to improve (or worsened), in terms of quality of life following ACT+. Additionally, we will conduct individual interviews with therapists who have delivered ACT+ as part of the SURECAN trial, to understand their experiences of delivering ACT+.

**Conclusions:**

Consistent with other qualitative protocols, this protocol is not registered. Instead, it is shared as a means of documenting ahead of time, how we are endeavouring to understand the ways in which a newly trialled talking therapy is received by patients and therapists, and how (if successful) it might be incorporated into the NHS.

## Introduction

This interview study will form part of the mixed methods process evaluation of the Survivors’ Rehabilitation Evaluation after Cancer (SURECAN) trial, which will be conducted following Medical Research Council guidance ^1^. The SURECAN trial is directed to people who have completed cancer treatment with curative intent. For example, some people with prostate cancer continue with long-term, ongoing maintenance treatment in the form of androgen suppression therapy. People receiving this type of maintenance treatment are eligible for recruitment into the SURECAN trial. In addition, people with certain haematological cancers are treated with the intention of long-term remission, which means they are never technically disease-free. This is why we have included within our eligibility definition “treated with curative intent/long-term remission”. For these reasons, we prefer to use the term ‘living with and beyond’ cancer. SURECAN is a multi-centre, pragmatic, individual participant randomised controlled trial of an intervention based on acceptance and commitment therapy (a talking therapy) supplemented by support for return to meaningful work and/or physical activity, according to the preferences of the individual study participant, known as ‘ACT+’.

ACT+ is personalised to participants and includes a range of theoretically informed interventions which target people's experience of symptoms, distress and quality of life. As part of the intervention, behavioural goals are not standardized. Instead, we are interested in understanding the full range of experiences and behaviours of participants in relation to their health. We do not standardise behaviour patterns. While quantitative research aims to standardise and limit variables under investigation, qualitative research (separate from the therapy under investigation) as undertaken in this study does not pre-specify what should be attended to, as it is designed to explore complex phenomena ^2^.

Trial participants receive up to eight one-hour sessions of ACT+ weekly or fortnightly delivered by telephone or online. Therapists attend an ACT+ training workshop delivered over two to three days, and receive regular supervision during the trial from an experienced cognitive behavioural therapist with extensive training in ACT. We have reported in more depth on the development and evaluation of the training programme ^3^. Additional information about the intervention will be available in the protocol for the main quantitative trial looking at the effectiveness and cost-effectiveness of the ACT+ intervention (to be published in the public domain in due course). The ACT+ intervention in addition to usual aftercare is compared to usual aftercare only, for patients living with and beyond cancer (SURECAN Trial IRAS: 260823 Protocol v3.0 06/02/2022). Trial participants comprise individuals who have completed treatment with curative intent for one of five cancer groups (breast, lower gastrointestinal, haematological, head and neck, urological) and are experiencing low quality of life as assessed by the Functional Assessment of Cancer Therapy: General scale (FACT-G) ^4^.

Importantly, instead of evaluating cancer types, we are evaluating the impact of acceptance and commitment therapy (ACT), which is an empirically supported trans-diagnostic psychological therapy for wide-ranging health conditions, including varying types of cancer ^5^. While psychological contexts vary broadly for different cancers and patient characteristics, the focus of acceptance and commitment therapy (ACT) is to help patients better adapt to the significant challenges they face, by promoting psychological flexibility, and helping them choose what they focus on, rather than invest energy in trying to suppress or ignore difficulties. Research suggests that ACT is helpful across a range of cancer types ^6^, although more research is needed to identify the features of the interventions and patient characteristics that could be used to improve results. Hence, the qualitative research proposed with diverse patients and types of cancers – where we will compare those who improve and do not improve - is crucial. Trial participants are recruited through participating hospital cancer clinics, and the ACT+ intervention is delivered by trained therapists working in either participating NHS Talking Therapies services in primary care mental health services or the charity sector.

## Purpose

This interview study addresses the ways in which participants believe they benefit from ACT+ (or not), and how the ACT+ intervention might best be implemented into routine National Health Service (NHS) care. The purpose of the study is to investigate the experience of ACT+ by different participants to understand how we can optimise the ACT+ intervention and its delivery (assuming the intervention is successful). In particular, we will focus on comparing our interview findings from those trial participants who improved and from those who failed to improve (or worsened), in terms of quality of life following ACT+. Improvement will be identified as an increase of more than 6 points on the FACT-G scale. This will only be determined for individual participants at the end of the study after data lock. Participants are sampled and interviewed by researchers who are blind to their change in FACT-G score. We anticipate our sample is large enough to meaningfully compare participants who appear to have improved and not improved on these criteria. The change or not in FACT-G will then be used to interpret the analysis. We will also capture the experience of therapists who delivered the ACT+ intervention.

We will investigate experiences of ACT+, and ACT+ delivery, in two parts:

In Part A we will conduct individual interviews with participants who have taken part in the active arm of the SURECAN trial to understand their experiences of engaging with and receiving ACT+, their perceptions of the impact of the therapy, and relevant contextual factors influencing these experiences.

In Part B we will conduct individual interviews with therapists who have delivered ACT+ as part of the SURECAN trial, to understand their experiences of delivering ACT+ to people who are living with and beyond cancer.

## lImportance and theoretical framework

We will draw on Normalisation Process Theory ^7^, a theory that focuses on how innovations are incorporated into systems like the NHS. This approach essentially means that in our lines of questioning both participants and therapists, we will ensure to cover specific contexts of the trial; coherence (i.e. how people make sense) of the approaches used; cognitive participation (how people think about the delivery of the innovation); collective action (what people do to deliver an innovation); and reflective monitoring (how people evaluate their contributions and/or the consequences of the trial). This will ensure we ask pertinent questions of both trial participants and therapists; that we elicit narratives in order to explore how trial participants subjectively appraise their experiences related to ACT+; and explore how to best integrate ACT+ into the NHS should the therapy prove useful ^8, 9^. This current qualitative study is particularly important because if the SURECAN trial is successful, and ACT+ is integrated into the NHS, it is critical to know more about why some patients benefit and others do not. This is so that ACT+ can be optimised to help the greatest number of patients possible in the NHS. When we used a similar study approach in a previous trial of Graded Exercise Therapy, we discovered factors linked to participant improvement (e.g. patient motivation, being able to tolerate an initial phase of no improvement) ^10^. Thus, this information allows practitioners to subsequently refine their treatment approach to address factors that will improve the likelihood of success.

## Preregistration

Currently, templates for preregistering qualitative protocols are generally not yet well developed for qualitative research (e.g. quantitative assumptions, templates not fitting qualitative research paradigms), especially given qualitative research involves ongoing iterative changes to study designs to respond to emerging insights in the field ^11^. There is still some work needed to ensure templates are suitable for qualitative preregistration ^12^, and for this reason we have not preregistered our protocol.

## Research questions

Our research questions for part A are:

1) What are the differences in treatment perceptions and experiences between those trial participants who improved and those who did not following ACT+?
2) Why might different kinds of participants do better than others with ACT+?
3) How do participants explain the influence of life contexts on their outcomes?
4) How can we optimise the ACT+ intervention and its delivery, with regard to future implementation?

Our research question for part B is:

1) How can we optimise the ACT+ intervention and its delivery, with regard to future implementation?
2) Why do therapists think different kinds of participants might do better than others with ACT+?

## Sample and recruitment for part A

### Eligibility criteria

The inclusion criteria are:

1. participant in intervention arm of trial
2. received at least four sessions of ACT+ (Note: Four sessions of ACT+ were considered the minimum ‘optimal dose’, where improvements generally take at least 4 sessions for those with common mental health problems who are likely to respond to short-term psychological treatments ^13^.)
3. no longer receiving ACT+

The exclusion criteria are:

1. did not give consent to be approached for an interview
2. more than 14 months since final ACT+ session

### Sampling

***Size of sample.*** We aim to recruit up to 30 participants randomised to the intervention arm of the trial. Our previous research has shown that we need to recruit at least 9 participants in each of the improvement and non-improvement groups to make useful comparisons between participants ^10^. As we will not know which group the participants fall into until after the trial is unblinded, we believe that a sample of up to 30 will ensure we have sufficient numbers in each group to be able to compare patients with improved and not improved ‘quality of life’ scores.

***Sampling strategy.*** We will conduct purposive sampling to obtain variation in participant characteristics. Dimensions of interest are cancer group, age, gender, and ethnic group (White, Black or Black British, Asian or Asian British, Mixed, Other), although other dimensions of interest may emerge iteratively.

### Recruitment

***Sample identification.*** A list of participants eligible for this study, and their demographic characteristics, will be extracted from the SURECAN trial database. Data extraction will take place while the trial is live.

From this list of eligible participants a sample of participants will be selected to approach for interview. This sample will be selected to provide variation in participant characteristics like cancer group, age, gender, and ethnicity. Where multiple participants share the same characteristics the selections from that group will be made randomly. Once participants have been approached for interview they will be removed from any future eligible participant lists.

The process of sample selection will be iterative, with the first sample chosen to provide overall diversity but assigning more weight to selecting a variety of different ‘cancer groups’ as far as possible, the aim being to identify a group of potential participants who have been treated for different cancers. We will not aim to identify equal numbers for each cancer group as it is likely that not everyone invited into this interview study will agree to participate. The trial statistician (CR) will work closely with the qualitative researcher (SD) to determine how many trial participants need to be identified in each sampling cycle.

When interviews have been conducted with individuals recruited from the first sample selected, information regarding their cancer group, age, gender, and ethnicity (available from the extracted data and confirmed with participants at the time of interview) will be collated by the qualitative researcher to produce an overview of the variation in the sample to date. This information on the make-up of the sample will be reviewed by the research team to determine which of the categories (our dimensions of interest) should receive more weight in the second sample selection in order to increase the variation in the sample. The need for any subsequent sample selection will be determined in a similar way. The need for any subsequent data extraction/s will depend on the number of participants recruited for interview from the samples selected (as described above) in relation to our target sample size of up to 30 interviewees.

**Figure 1.**
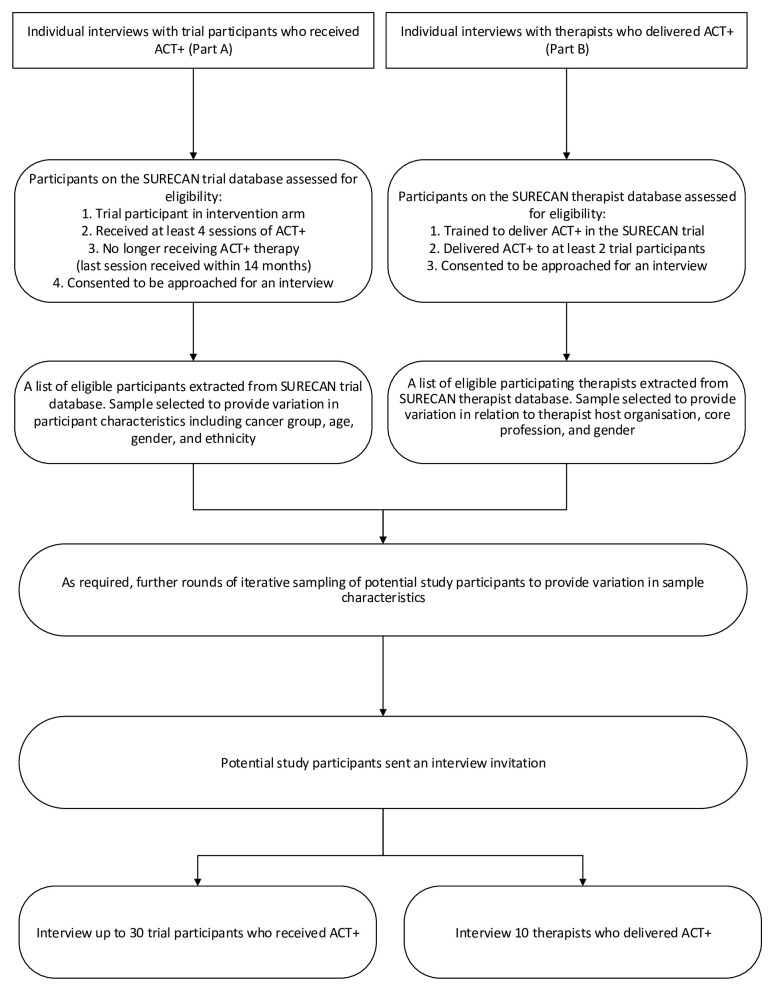
Study flow diagram.

The qualitative researcher will liaise with the interview study lead (DR), the trial manager (IK), the trial statistician (CR), and the research team at regular intervals to review how the process of forming the sample is progressing and to agree the timing and objectives of any subsequent data extraction/s. See Figure 1 (Study flow diagram) for an illustration of how participants for Part A will be identified.

***Consent.*** Consent to be approached about post-therapy interviews was sought at the time that consent to participate in the trial was obtained.

Initial contact will be made by post or email. The qualitative researcher will send potential participants, by post or electronically, an invitation pack containing an invitation letter, study information sheet, consent (or e-consent) form, and prepaid envelope (where appropriate) to return the consent form. The invitation letter will explain that the researcher can be contacted for further information and to address any queries. Between seven and 10 days after posting the invitation pack (and if the consent form has not been returned), the qualitative researcher will follow up with a telephone call to discuss the individual’s potential participation and answer any questions they have about the study. Subsequent to the invitation letter, a total of up to three phone attempts, and one email attempt (if appropriate) will be made to speak/communicate with the potential participant over a 30-day period. No further attempt will be made to make contact.

The researcher will explain to potential participants that although invited to participate in an interview, their involvement is entirely voluntary, and they can stop the interview at any time, no questions asked.

***Patient and Public Involvement.*** Two Patient and Public Involvement (PPI) representatives were grant co-applicants for the SURECAN research programme. Along with other PPI representatives, they have been actively involved in SURECAN through regular programme management meetings as well as specific PPI meetings, and through participation in reviewing and commenting on patient-facing materials used in the ACT+ therapy sessions. Five SURECAN PPI representatives reviewed the invitation letter/email, the study information sheet and the interview schedule for trial participants in this qualitative study. Interim findings from this qualitative study will be presented to the SURECAN PPI representatives for debate.

## Sample and recruitment for part B

### Eligibility criteria

The inclusion criteria are:

1. therapist trained to deliver ACT+ in the SURECAN trial
2. delivered ACT+ sessions to at least two trial participants

The exclusion criterion is:

1. did not give consent to be approached for an interview

### Sampling

***Size of sample trial and strategy.*** We aim to recruit 10 therapists participating in the SURECAN trial, out of around 25-30 therapists who are participating in the SURECAN trial. This sample size allows us to purposefully sample ^14^, to improve rigour by including a range of views according to differences in host organisations, core professions, levels of experience and genders.

### Recruitment

***Sample identification.*** A list of therapists eligible for this study, and details of their host organisation, core profession, and gender, will be extracted from the SURECAN therapist database. From this list, a purposive sample selected to provide variation in the dimensions of interest will be approached for interview. See Figure 1 (Study flow diagram) for an illustration of how participants for Part B will be identified.

***Consent.*** Consent to be approached about post-intervention delivery interviews was sought at the time that consent to participate in the trial was obtained.

Initial contact will be made by email. The qualitative researcher will send an invitation pack (containing an invitation letter, study information sheet, and e-consent form) to potential participants electronically. The invitation letter will explain that the researcher can be contacted for further information and to address any queries. Between seven and 10 days after sending the invitation pack, (and if the consent form has not been returned), the qualitative researcher will follow up with an email, to remind the therapist about the invitation pack, ask if they have any questions about the study, and offer to speak on the phone at a convenient time to discuss their possible participation. Subsequent to the invitation letter, up to five reminders via email and/or phone will be made during a period of 30 days. No further attempt will be made to make contact.

The researcher will explain to potential participants that although invited to participate in an interview, their involvement is entirely voluntary, and they can stop the interview at any time, no questions asked.

## Participant involvement

Participants in this interview study (Parts A and B) will take part in a one-off, individual semi-structured interview, conducted either by telephone or via a data protection-compliant online platform (Skype or Microsoft Teams), whichever is their preference. Interviews will last for 40 to 60 minutes.

## Data collection

The use of a semi-structured interview approach will i) allow us to address the same topics in each set of interviews and in so doing, generate comparable data about participants’ experience of receiving or delivering the ACT+ intervention, and ii) provide sufficient flexibility within the interviews to enable participants to highlight their concerns and elaborate on particular aspects in their accounts ^15^.

While the interview guides were not pilot tested, they were developed collaboratively by the study team, drawing on: members’ wide expertise, and interview guides we had developed for the SURECAN pre-pilot study (a small test-run of ACT+, followed by individual interviews with participating patients and ACT+-trained therapists). We also drew from interview guides used in a similar study comparing trial participants’ experiences of improvement and non-improvement for an intervention ^10^. Additionally, the interview guide for trial participants was reviewed by five of the SURECAN study PPI representatives. Finally, in qualitative research, it should be noted that interview guides are seen as ‘a work in progress’ ^16^. It is recommended that guides be further refined periodically as the researcher conducts interviews in the field, as emerging insights suggest how topics should be refined or added. Topics for interviews with trial participants (Part A) will include the decision to take part in the SURECAN trial, expectations of the therapy, concerns about the therapy, understanding of ACT+, barriers and facilitators to ACT+, engagement in the ACT+ sessions, use of the ACT+ Participant Handbook, perceived impact of the therapy, why ACT+ worked/did not work, anything important going on at the time of ACT+, challenges emerging after completing the course of therapy. The interviewer will specifically enquire about issues highlighted by participants as relating to ACT+ and/or its effectiveness, including but not limited to, the anatomical site of cancer, comorbidities, functional implications, gender and patient age.

Topics for interviews with therapists (Part B) will include working with the client group (people living with and beyond cancer), delivering the therapy in a trial context, delivery of ACT+ sessions, use of the ACT+ Therapist Manual, ending the therapy, perceived value of ACT+ for the client (their allocated trial participant).

## Data analysis and data management

### Data analysis

Interviews will be audio-recorded and transcribed *verbatim* by a professional transcribing service with which the university has an agreement, including to treat audio recordings and the resultant transcripts as strictly confidential. The qualitative researcher will review transcripts against the audio recordings to correct any errors and remove any identifying information.

Data will be managed in the qualitative data analysis software environment NVivo. All transcripts, once checked for accuracy and anonymised, will be uploaded to NVivo and coded. A close thematic analysis of the data will be conducted to identify ‘repeated patterns of meaning’ ^17^. The analysis will incorporate a ‘constant comparison’ approach, to ensure that relevant data are compared with similar data systematically ^18^.

***Blinding.*** Initially, analysis of the trial participant interview data set will be conducted using baseline data only. When the SURECAN trial has been completed and we are unblinded to the study outcomes, we will conduct further analysis, comparing interview findings from participants who improved and those who did not improve following ACT+. It will not be possible to ensure equal numbers of trial participants who improved versus those who did not. Nor will it be possible to purposively sample trial participants who improved versus those who did not, as the researchers will be blinded to treatment outcome during the sampling phase.

The data extraction to identify eligible participants will be conducted by a statistician independent to the SURECAN trial to ensure the SURECAN trial statisticians remain blind to treatment group allocation of participants.

### Data management

Information related to participants will be kept confidential and managed in accordance with the General Data Protection Regulation (GDPR), NHS Caldicott Principles, The Research Governance Framework for Health and Social Care, and the conditions of Research Ethics Committee Approval.

The study information sheet will set out arrangements relating to confidentiality, security, storage and accessibility of data only to the study team.

The signed consent forms will kept in a locked cabinet at Queen Mary, University of London, accessible by authorised study staff only. All data collected will be fully anonymised by a unique participant ID. For telephone interviews, the qualitative researcher will use an encrypted digital audio-recorder to record the interview. The recording will be downloaded onto a secure and encrypted USB storage device immediately following the interview. For interviews conducted using a secure online calling platform, the recording function of the secure platform will be used to record the interview. The recording will be downloaded onto a secure and encrypted USB storage device immediately following the interview. Encrypted USBs are kept in a locked cabinet in a locked room.

A copy of the recordings will be downloaded onto an encrypted USB storage device and sent securely to a professional transcriber for transcription. The transcriber will upload the transcribed documents onto the USB storage device and return it securely to the study team.

All recording file data will be uploaded onto a dedicated folder on the secure virtualised environment at the Barts Cancer Centre (BCC) at Queen Mary, University of London, and deleted from the digital recorder and, after analysis, the encrypted storage devices. The folders where the data are stored will be accessible only to the appropriate members of the SURECAN study team.

## Ethical and regulatory considerations

### Research ethics approval

A favourable opinion from a Health Research Authority Research Ethics Service for the study protocol, consent forms, invitation letters and participant information sheets has been obtained ( **IRAS Number** 314406, **REC Number** 22/SW/0157).

### Ethical considerations

The Co-Chief Investigators will ensure that the study is carried out in accordance with the ethical principles in the Research Governance Framework for Health and Social Care, Second Edition, 2005, and its subsequent amendments as applicable together with applicable legal and regulatory requirements.

The informed consent process has been described in the consent section above. Consent materials comprise a study information sheet, an invite letter, and a consent form. We have made a particular effort to use clear, accessible language in these documents and have received advice on them from our study patient advisors. The information sheet covers the purpose of the study, why potential participants have been approached to take part and what would it mean for them if they chose to participate, the benefits and risks of participation, assurance that participation is voluntary and that withdrawal from the study can be at any time, the type of data collection, data storage, confidentiality and security, who the study is funded and sponsored by, who reviewed the study, and whom to contact for further information. Participants will be given a copy of their signed consent form at the time of their recruitment into the study.

There is potential for patient participants to become upset about their situation or their condition. If an interviewee becomes distressed, the interviewer will stop the interview and will stay with the participant while they recover, and check in with such participants by telephone in the days subsequent to the interview. Information as to how they can seek further help will be offered to participants.

## Sponsorship and indemnity

Queen Mary University of London will be the study sponsor. The sponsorship will be given on the basis of meeting the ‘Conditions of sponsorship’ which means that the research should be conducted and managed as per the Research Governance Framework for Health and Social Care 2005 and/or the Medicines for Human Use (Clinical Trials) Regulations 2004.

Queen Mary University of London has a no-fault indemnity insurance policy for research participants. These compensation arrangements apply where harm is caused to a participant that would not have occurred if they had not taken part in the study. These arrangements do not affect participants’ rights to pursue a claim through legal action.

## Data Availability

No data are associated with this article.

## References

[1] Moore GF Audrey S Barker M : Process evaluation of complex interventions: Medical Research Council guidance. *BMJ.* 2015;350: h1258. 10.1136/bmj.h1258 25791983 PMC4366184

[2] Peshkin A : Understanding Complexity: A Gift of Qualitative Inquiry. *Anthropol Educ Q.* 1988;19(4):416–424. Reference Source

[3] Moschopoulou E Brewin D Ridge D : Evaluating an interactive acceptance and commitment therapy (ACT) workshop delivered to trained therapists working with cancer patients in the United Kingdom: a mixed methods approach. *BMC Cancer.* 2022;22(1): 651. 10.1186/s12885-022-09745-4 35698089 PMC9195438

[4] Cella DF Tulsky DS Gray G : The Functional Assessment of Cancer Therapy scale: development and validation of the general measure. *J Clin Oncol.* 1993;11(3):570–9. 10.1200/JCO.1993.11.3.570 8445433

[5] Dindo L Van Liew JR Arch JJ : Acceptance and Commitment Therapy: A Transdiagnostic Behavioral Intervention for Mental Health and Medical Conditions. *Neurotherapeutics.* 2017;14(3):546–553. 10.1007/s13311-017-0521-3 28271287 PMC5509623

[6] González-Fernández S Fernández-Rodríguez C : Acceptance and Commitment Therapy in Cancer: Review of Applications and Findings. *Behav Med.* 2019;45(3):255–269. 10.1080/08964289.2018.1452713 29558259

[7] Murray E Treweek S Pope C : Normalisation process theory: a framework for developing, evaluating and implementing complex interventions. *BMC Med.* 2010;8(1): 63. 10.1186/1741-7015-8-63 20961442 PMC2978112

[8] Greenhalgh T Hurwitz B : Why study narrative? *BMJ.* 1999;318(7175):48–50. 10.1136/bmj.318.7175.48 9872892 PMC1114541

[9] Hurwitz B Greenhalgh T Skultans V : Narrative research in health and illness.BMJ Books,2008. Reference Source

[10] Cheshire A Ridge D Clark L : Guided graded Exercise Self-help for chronic fatigue syndrome: patient experiences and perceptions. *Disabil Rehabil.* 2020;42(3):368–377. 10.1080/09638288.2018.1499822 30325677

[11] Branney PE Brooks J Kilby L : Three steps to open science for qualitative research in psychology. *Soc Personal Psychol Compass.* 2023;17(4): e12728. 10.1111/spc3.12728

[12] Haven TL Errington TM Gleditsch KS : Preregistering Qualitative Research: A Delphi Study. *Int J Qual Methods.* 2020;19: 1609406920976417. 10.1177/1609406920976417

[13] Robinson L Delgadillo J Kellett S : The dose-response effect in routinely delivered psychological therapies: A systematic review. *Psychother Res.* 2020;30(1):79–96. 10.1080/10503307.2019.1566676 30661486

[14] Campbell S Greenwood M Prior S : Purposive sampling: complex or simple? Research case examples. *J Res Nurs.* 2020;25(8):652–661. 10.1177/1744987120927206 34394687 PMC7932468

[15] McIntosh MJ Morse JM : Situating and constructing diversity in semi-structured interviews. *Glob Qual Nurs Res.* 2015;2: 2333393615597674. 10.1177/2333393615597674 28462313 PMC5342650

[16] Adams WC : Conducting Semi-Structured Interviews.In: *Handbook of Practical Program Evaluation.*K.E. Newcomer, H.Hatry, and J.S. Wholey, Editors. Jossey-Bass.2015;492–505. 10.1002/9781119171386.ch19

[17] Braun V Clarke V : Using thematic analysis in psychology. *Qual Res Psychol.* 2006;3(2):77–101. 10.1191/1478088706qp063oa

[18] Glaser BG : The constant comparative method of qualitative analysis. *Social Problems.* 1965;12(4):436–445. 10.2307/798843

