# Supplementary material for: Qualitative study of experience of acceptance and commitment therapy (ACT+) amongst Survivors' Rehabilitation Evaluation after Cancer (SURECAN) trial participants and therapists: A protocol
Source: NIHR Open Res. 2024 Jan 3;3:24. Originally published 2023 May 12. [Version 2] doi: 10.3310/nihropenres.13382.2 (PMC11319895; doi:10.3310/nihropenres.13382.2)
Supplement: Peer review history

# Reviewer report: Reviewer response for version 2

**DOI:** 10.3310/nihropenres.14686.r31012

**Published:** 2024-03-06

**Authors:** Daniel Sat-Muñoz

**Affiliations:** 1 Cuerpo Académico UDG-CA-874 Ciencias Morfológicas en el Diagnóstico y Tratamiento de la Enfermedad, Universidad de Guadalajara, Guadalajara, Jalisco, Mexico; 2 Departamento de Oncología Quirúrgica, UMAE Hospital de Especialidades del Centro Médico Nacional de Occidente. Instituto Mexicano del Seguro Social, Guadalajara, Jalisco, Mexico

The authors have realized some modifications, and they are using them to justify the number of studied subjects (I understand that a qualitative study does not apply a sample size); however, as clinicians, we know that patients will develop different psychological affectations according to the type of cancer, the location, and clinical stage. I suggest centering the research only on one or two types of cancer.

 Some punctuality issues are: 

 1. The sample size is too small considering the number of oncologic pathologies in the protocol, considering too, the mixture of symptoms, the different types of treatment and their psychological impact, and the gender of patients, among other differences. 

 2. Biologically, prostate cancer is different from hematological malignancies and affects other aspects of the patient's life, and this is only an example. 

 3. In a complex phenomenon, focusing on one malignant neoplasm or a maximum of two is easy. This situation permits the identification of the phenomena in a complex disease such as cancer.

Is the study design appropriate for the research question?

Partly

Is the rationale for, and objectives of, the study clearly described?

Partly

Are sufficient details of the methods provided to allow replication by others?

Partly

Are the datasets clearly presented in a useable and accessible format?

Partly

Reviewer Expertise:

Surgical Oncology and its impact in Health Related Quality of Life, functionality and Nutritional State

I confirm that I have read this submission and believe that I have an appropriate level of expertise to confirm that it is of an acceptable scientific standard, however I have significant reservations, as outlined above.

# Reviewer report: Reviewer response for version 2

**DOI:** 10.3310/nihropenres.14686.r31013

**Published:** 2024-02-26

**Authors:** Rebecca L. Gould

**Affiliations:** 1 Division of Psychiatry, University College London, London, England, UK

The authors have addressed my comments well and so I approve this version.

Is the study design appropriate for the research question?

Yes

Is the rationale for, and objectives of, the study clearly described?

Yes

Are sufficient details of the methods provided to allow replication by others?

Partly

Are the datasets clearly presented in a useable and accessible format?

Not applicable

Reviewer Expertise:

Psychological therapies, clinical trials

I confirm that I have read this submission and believe that I have an appropriate level of expertise to confirm that it is of an acceptable scientific standard.

# Reviewer report: Reviewer response for version 1

**DOI:** 10.3310/nihropenres.14513.r29909

**Published:** 2023-08-18

**Authors:** Daniel Sat-Muñoz

**Affiliations:** 1 Cuerpo Académico UDG-CA-874 Ciencias Morfológicas en el Diagnóstico y Tratamiento de la Enfermedad, Universidad de Guadalajara, Guadalajara, Jalisco, Mexico; 2 Departamento de Oncología Quirúrgica, UMAE Hospital de Especialidades del Centro Médico Nacional de Occidente. Instituto Mexicano del Seguro Social, Guadalajara, Jalisco, Mexico

It is a well-written protocol; however, this reviewer observed some methodologic design details related to clinical and oncologic issues: 

1. The protocol is qualitative about of experience of acceptance and commitment therapy (ACT+) amongst Survivors’ Rehabilitation Evaluation after Cancer (SURECAN). However, the protocol does not specify which type of cancer will be evaluated by the intervention. 
2. The psychological context varies broadly in different cancers and different patients’ characteristics (women with mastectomy, men with prostate cancer and erectile dysfunction, patients with head and neck cancer and mutilating face surgeries, among others)
3. How will the researchers standardize the behavior patterns in patients with various cancers, genders, and conditions?
4. Despite the qualitative nature of the protocol is essential to the capacity to distinguish the differential response patterns in the patients according to the anatomical site of cancer, the functional implications, the gender, and the role function affected or not by the tumor or patient age, and the influence of the aging or comorbidities.
5. Even the therapeutic team will develop a differential behavior in front of different types of cancer, so it is imperative to delimitate the specific cancer and even the clinical stage or the treatment for the cancer. 
6. Authors must decide on anatomic site, histologic characteristics, clinical stage, and treatment modality and evaluate according to specific selection criteria, considering clinical and disease-specific characteristics.

Is the study design appropriate for the research question?

Partly

Is the rationale for, and objectives of, the study clearly described?

Partly

Are sufficient details of the methods provided to allow replication by others?

Partly

Are the datasets clearly presented in a useable and accessible format?

Partly

Reviewer Expertise:

Surgical Oncology and its impact in Health Related Quality of Life, functionality and Nutritional State

I confirm that I have read this submission and believe that I have an appropriate level of expertise to state that I do not consider it to be of an acceptable scientific standard, for reasons outlined above.

# Response

**Published:** 2023-12-14

**Authors:** Damien Ridge

**Affiliations:** University of Westminster, UK

**Dear Professor Sat-Muñoz**

**Many thanks for noting our protocol is "well-written". We have carefully considered and responded to your points in turn below:**

  

1. **protocol is qualitative about of experience of acceptance and commitment therapy (ACT+) amongst Survivors’ Rehabilitation Evaluation after Cancer (SURECAN). However, the protocol does not specify which type of cancer will be evaluated by the intervention.**

We are not evaluating cancer types *per se*. However, we do list the details of the five cancer types included in the study in the ‘Introduction’ section of the protocol, specifically, we include breast, lower gastrointestinal, haematological, head and neck, and urological. Instead of evaluating cancer types, we are evaluating the impact of acceptance and commitment therapy (ACT), which is an empirically supported *trans-diagnostic* psychological therapy for wide-ranging health conditions, including varying types of cancer (Dindo et al., 2017). We have added this information:

*“Importantly, instead of evaluating cancer types, we are evaluating the impact of acceptance and commitment therapy (ACT), which is an empirically supported trans-diagnostic psychological therapy for wide-ranging health conditions, including varying types of cancer* *(Dindo et al., 2017)* *.”*

  

1. **The psychological context varies broadly in different cancers and different patients’ characteristics (women with mastectomy, men with prostate cancer and erectile dysfunction, patients with head and neck cancer and mutilating face surgeries, among others)**

This is an important point made by the reviewer. The focus of acceptance and commitment therapy (ACT) is to help patients adapt more productively to these very significant challenges as outlined by the reviewer, by increasing psychological flexibility and assisting patients to choose what to focus on, rather than investing energy in trying to suppress or ignore difficulties. Research suggests that ACT is helpful across a range of cancer types, although more is needed to understand the features of the interventions and patient characteristics that could improve results (González-Fernández & Fernández-Rodríguez, 2019). Hence, the qualitative research proposed with diverse patients and types of cancers – where we will compare those who improve and do not improve - is needed. We have added the following note:

*“While psychological contexts vary broadly for different cancers and patient characteristics, the focus of* *acceptance and commitment therapy* *(ACT) is to help patients better adapt to the significant challenges they face, by increasing psychological flexibility, and helping them choose what they focus on, rather than invest energy in trying to suppress or ignore difficulties. Research suggests that ACT is helpful across a range of cancer types* *(González-Fernández & Fernández-Rodríguez, 2019)* *, although more research is needed to identify the features of the interventions and patient characteristics that could be used to improve results. Hence, this qualitative interviews research proposed with diverse patients and types of cancers – where we will compare those who improve and do not improve - is crucial.”*

1. **How will the researchers standardize the behaviour patterns in patients with various cancers, genders, and conditions?**
**  **

ACT+ is personalised and includes a range of interventions which target people's experience of symptoms, distress and improvement of Quality of Life. We do not, as part of ACT+  standardise behavioural goals. We are instead interested in understanding the full range of different experiences and behaviours of participants in relation to ACT+, so it is not useful to focus on standardising behaviour patterns. While quantitative research aims to standardise and limit variables under investigation, qualitative research on the other hand does not pre-specify in this way what should be attended to, as it is designed to explore complex phenomena (Peshkin, 1988). The protocol for the main quantitative trial looking at the effectiveness and cost-effectiveness of the ACT+ intervention will be available in the public domain shortly. We have added the following note:

*“ACT+ is personalised to participants and includes a range of theoretically informed interventions which target people's experience of symptoms, distress and quality of life. As part of the intervention, behavioural goals are not standardized.* *Instead, we are interested in understanding the full range of different experiences and behaviours of participants in relation to their health. We do not standardise behaviour patterns. While quantitative research aims to standardise and limit variables under investigation, qualitative research (separate from the therapy under investigation) as undertaken in this study does not pre-specify what should be attended to, as it is designed to explore complex phenomena* *(Peshkin, 1988)* *.”*

1. **Despite the qualitative nature of the protocol is essential to the capacity to distinguish the differential response patterns in the patients according to the anatomical site of cancer, the functional implications, the gender, and the role function affected or not by the tumor or patient age, and the influence of the aging or comorbidities.**
**  **

As above, qualitative research is able to explore this complexity. We will take on board the reviewer's comments here by ensuring we enquire about these topics in the interview where relevant, i.e. where they are raised by participants as important to understanding ACT+, including anatomical site of cancer, comorbidities, functional implications, gender role or patient age. We have added the following note:

*“The interviewer will specifically enquire about issues highlighted by participants as relating to ACT+ and/or its effectiveness, including but not limited to, the* *anatomical site of cancer, comorbidities, functional implications, gender* *and* *patient age* *.”*

1. **Even the therapeutic team will develop a differential behavior in front of different types of cancer, so it is imperative to delimitate the specific cancer and even the clinical stage or the treatment for the cancer.**

As above, we will specifically enquire about these topics where relevant. 

1. **Authors must decide on anatomic site, histologic characteristics, clinical stage, and treatment modality and evaluate according to specific selection criteria, considering clinical and disease-specific characteristics.**

As above, as this is not quantitative research, we do not standardise our approach.

# Reviewer report: Reviewer response for version 1

**DOI:** 10.3310/nihropenres.14513.r30025

**Published:** 2023-08-18

**Authors:** Ana Joaquim

**Affiliations:** 1 ONCOMOVE, Associação de Investigação de Cuidados de Suporte em Oncologia (AICSO), Vila Nova de Gaia, Portugal; 2 European Organisation for Research and Treatment of Cancer, Brussels, Brussels, Belgium

This paper is the protocol of a preplanned secondary analysis of the trial SURECAN, on a qualitative study about the psychological intervention ACT+ among participants and the therapists who deliver the intervention. Should the intervention be proven successful by the main trial, with this secondary analysis the authors are aiming to assess ways to incorporate it into the NHS.

 It's a well-designed study based on important practical and pragmatic questions to be answered.

 There are, however, some questions I would like to be addressed before its publication:

 1. The participants of the SURECAN trial are described as people living with and beyond cancer. However, I understood that the trial is directed to people who were treated for cancer and are disease-free, meaning the population of the SURECAN should be described as "people living beyond cancer" only. If I understood correctly, people living with cancer are  excluded.

 2. The authors justify the absence of registry of the protocol with the qualitative nature of the study. I suggest to rethink this decision or, in alternative, to expand on the justification, as there is bibliography in favor of registering qualitative studies ^1^.

 3. Introduction section: 

 3.1. I feel that information on the importance of the topic is missing. Why do the authors believe that this qualitative study is important and useful? What is the background? What has been done in the past? 

 3.2. The five cancer groups are not consistent with what is advertised in the site of the trial - is it urological (as it is in the paper) or prostate (as it is stated in the site)?

 3.3. The first and only time the acronym IAPT appears, it is not explained

 4. The sample size of both parts A and B are not justified at all. I strongly advise to explain why is this the sample size, if there were any assumptions and which were they?

Is the study design appropriate for the research question?

Yes

Is the rationale for, and objectives of, the study clearly described?

Yes

Are sufficient details of the methods provided to allow replication by others?

Partly

Are the datasets clearly presented in a useable and accessible format?

Not applicable

Reviewer Expertise:

Supportive care in Oncology, physical exercise and cancer, health-related quality of life

I confirm that I have read this submission and believe that I have an appropriate level of expertise to confirm that it is of an acceptable scientific standard, however I have significant reservations, as outlined above.

## References

[1] : Preregistering qualitative research. *Accountability in Research* .2019;26(3) : 10.1080/08989621.2019.1580147 229-244 10.1080/08989621.2019.1580147 30741570

# Response

**Published:** 2023-12-14

**Authors:** Damien Ridge

**Affiliations:** University of Westminster, UK

**Dear Professor** **Joaquim**

**Many thanks for noting our protocol represents “a well-designed study based on important practical and pragmatic questions to be answered.”**

**We have addressed your questions in turn below, and believe that they have helped us to refine and clarify the manuscript:**

 1. **The participants of the SURECAN trial are described as people living with and beyond cancer. However, I understood that the trial is directed to people who were treated for cancer and are disease-free, meaning the population of the SURECAN should be described as “people living beyond cancer” only. If I understood correctly, people living with cancer are excluded.**

 We have added the following clarification:

*“The SURECAN trial is directed to people who have completed cancer treatment with curative intent. For example, some people with prostate cancer continue with long-term, ongoing maintenance treatment in the form of androgen suppression therapy. People receiving this type of maintenance treatment are eligible for recruitment into the SURECAN trial. In addition, people with certain haematological cancers are treated with the intention of long-term remission, which means they are never technically disease-free. This is why we have included within our eligibility definition, “treated with curative intent/long-term remission”. For these reasons we prefer the term ‘living with and beyond’ cancer.”*

 2. **The authors justify the absence of registry of the protocol with the qualitative nature of the study. I suggest to rethink this decision or, an alternative, to expand on the justification, as there is bibliography in favor of registering qualitative studies** **^1^** **.**

 We have added the following text to the protocol:

*“Currently, templates for preregistering qualitative protocols are generally not yet well developed for qualitative research (e.g. quantitative assumptions, templates not fitting qualitative research paradigms), especially given qualitative research involves ongoing iterative changes to study designs to respond to emerging insights in the field* *(Branney et al., 2023)* *. There is still some work needed to ensure templates are suitable for qualitative preregistration* *(Haven et al., 2020)* *, and for this reason we have not preregistered our protocol.”*

 3. **Introduction section:** 

 3.1. **I feel that information on the importance of the topic is missing. Why do the authors believe that this qualitative study is important and useful? What is the background? What has been done in the past?** 

 We have added the following text:

*“This* *current qualitative* *study is* *particularly* *important because if the* *SURECAN* *trial is successful, and ACT+ is integrated into the NHS, it is critical to know more about why some patients benefit and others do not. This is so that ACT+ can be optimised to help the most patients in the NHS. When we have used* *a similar* *study approach in a previous trial of Graded Exercise Therapy, we discovered factors linked to participant improvement (e.g.* *patient* *motivation,* *being able to* *tolerat* *e* *an initial phase of no improvement)* *(Cheshire et al., 2020)* *. Thus, this* *allows practitioners to* *subsequently* *refine their treatment* *approach to address factors that will improve the likelihood of success* *.”*

 3.2. **The five cancer groups are not consistent with what is advertised in the site of the trial - is it urological (as it is in the paper) or prostate (as it is stated in the site)?**

 Urological cancer, as stated in the paper, is correct. At the time that the SURECAN website went live, prostate cancer was one of the five cancer groups. During the pilot study, we broadened the eligibility criteria so as to include people who had been treated for either bladder cancer or renal cancer. Thus, the cancer group name was changed from ‘prostate’ to ‘urological’, and the SURECAN website has been updated to reflect this change, see: https://surecanstudy.qmul.ac.uk

 3.3. **The first and only time the acronym IAPT appears, it is not explained**

 IAPT stands for Improving Access to Psychological Therapies. Since January 2023, IAPT services have a new name: “NHS Talking Therapies” ( https://www.england.nhs.uk/blog/whats-in-a-name-nhs-talking-therapies-for-anxiety-and-depression-the-new-name-for-iapt-services/). We have amended the protocol and replaced “IAPT services” with “NHS Talking Therapies services”.

 4. **The sample size of both parts A and B are not justified at all. I strongly advise to explain why is this the sample size, if there were any assumptions and which were they?**

 We have added the following text to address part A:

*“Our previous research has shown that we need to recruit at least 9 participants in each of the improvement and non-improvement groups to make useful comparisons between participants* *(Cheshire et al., 2020)* *. As we will not know which group the participants fall into until after the trial is unblinded, we estimate that a sample of* *up to* *30 will ensure we have sufficient numbers in each group to be able* *to* *compare* *patients with* *improved and not improved ‘quality of life’ scores.”*

 As stated for part B:

 “ *We aim to recruit 10 therapists participating in the SURECAN trial, out of* *around 25-30 therapists who are participating in the SURECAN trial. This sample size allows us to purposefully sample* *(Campbell et al., 2020)* *, to improve rigour by including a range of views according to differences in host organisations, core professions, levels of experience and genders*.”

# Reviewer report: Reviewer response for version 1

**DOI:** 10.3310/nihropenres.14513.r29292

**Published:** 2023-07-20

**Authors:** Rebecca L. Gould

**Affiliations:** 1 Division of Psychiatry, University College London, London, England, UK

This is a well-written protocol for an interesting qualitative interview study which constitutes part of a mixed methods process evaluation of the SURECAN trial. The SURECAN trial is a pragmatic, parallel, single blind, two-arm randomised controlled trial of Acceptance and Commitment Therapy plus support to return to work and/or physical activity and usual aftercare (ACT+) for improving quality of life in people who are living with and beyond cancer in comparison to usual aftercare alone. Up to 30 people who are living with and beyond cancer, who have been randomly allocated to the ACT+ arm, will be interviewed, as well as up to 10 therapists involved in intervention delivery. Data will be analysed using thematic analysis. The protocol would benefit from further clarification of the following issues:

1. How improvement vs. no improvement will be defined in relation to QoL following ACT+. This needs to be specified *a priori* in order to avoid potential bias in qualitative data analysis in relation to research question 1 for Part A.
2. The justification as to why research question 2 for Part A will only be explored in trial participants and not therapists.
3. What the ACT+ intervention comprises as very little information is provided about it (e.g. brief information about the number of sessions, delivery format, how therapists are trained and supervised, etc).
4. The reason as to why one of the inclusion criteria for trial participants is having "received at least four sessions of ACT+" (e.g. because at least four sessions is considered a sufficient dose of ACT+).
5. It would be useful to highlight that it will not be possible to ensure equal numbers of trial participants who improved vs. those who did not, nor will it be possible to purposively sample trial participants who improved vs. those who did not as the researchers will be blinded to treatment outcome.
6. The justification for a sample size of 10 therapists.
7. Whether the interview guides will be pilot tested prior to recruitment (and if not, why not).
8. How Patient and Public Involvement members will be involved in the qualitative study.
9. Whether specific reporting guidelines will be used when reporting the qualitative study, such as the consolidated criteria for reporting qualitative research.

Is the study design appropriate for the research question?

Yes

Is the rationale for, and objectives of, the study clearly described?

Yes

Are sufficient details of the methods provided to allow replication by others?

Partly

Are the datasets clearly presented in a useable and accessible format?

Not applicable

Reviewer Expertise:

Psychological therapies, clinical trials

I confirm that I have read this submission and believe that I have an appropriate level of expertise to confirm that it is of an acceptable scientific standard, however I have significant reservations, as outlined above.

# Response

**Published:** 2023-12-14

**Authors:** Damien Ridge

**Affiliations:** University of Westminster, UK

**Dear Professor Gould,**

**Many thanks for noting our protocol “well-written" and "an interesting qualitative interview study”, as well as for your helpful comments aimed at improving the manuscript. We have further clarified the manuscript by addressing each of your points in turn as below:**

1. **How improvement vs. no improvement will be defined in relation to QoL following ACT+. This needs to be specified *a priori* in order to avoid potential bias in qualitative data analysis in relation to research question 1 for Part A.**

We have added the following clarification to the manuscript:

*“Improvement will be identified as an increase of more than 6 points on the FACT-G scale.  This will only be determined for individual participants at the end of the study after data lock. Participants are sampled and interviewed with the researchers blind to their change in FACT-G score. We anticipate our sample is large enough to meaningfully compare participants who appear to have improved and not improved on these criteria. The change or not in FACT-G will then be used to interpret the analysis.”*

  

1. **The justification as to why research question 2 for Part A will only be explored in trial participants and not therapists.**

The study was designed such that research questions 1 and 2 (Part A) would be addressed at the end of the trial when we will be unblinded to treatment outcomes and can conduct further analyses, comparing interview findings from participants who improved and those who did not improve following ACT+. Addressing research question 2 (Part A) in interviews by gathering the views of therapists is also worth doing, and we have added this to the protocol.

1. **What the ACT+ intervention comprises, as very little information is provided about it (e.g. brief information about the number of sessions, delivery format, how therapists are trained and supervised, etc).**

We have added the following information to the protocol:

*“Trial participants receive eight one-hour sessions of ACT+ weekly or fortnightly delivered by telephone or online. Therapists attend an ACT+ training workshop delivered over two to three days and receive regular supervision during the trial from an experienced cognitive behavioural therapist with extensive training in ACT. We have reported in more depth on the development and evaluation of the training* *(Moschopoulou et al., 2022)* *. Additional information about the intervention will be available in* *the protocol for the main quantitative trial looking at the effectiveness and cost-effectiveness of the ACT+ intervention, to be published in the public domain in due course.”*

1. **The reason as to why one of the inclusion criteria for trial participants is having "received at least four sessions of ACT+" (e.g. because at least four sessions is considered a sufficient dose of ACT+).**

As the reviewer suggests, we have included at least four sessions of ACT+, as this is considered the minimum ‘optimal dose’ of routine psychotherapies according to a recent systematic review (Robinson et al., 2020), which we have now explained in the protocol:

*“Four sessions of ACT+ were considered the minimum ‘optimal dose’, since research shows improvements generally take at least 4 sessions for those with common mental health problems who are likely to respond to short-term psychological treatments (Robinson et al., 2020).’*

1. **It would be useful to highlight that it will not be possible to ensure equal numbers of trial participants who improved vs. those who did not, nor will it be possible to purposively sample trial participants who improved vs. those who did not as the researchers will be blinded to treatment outcome.**

We have added this note to the protocol:

*“* *It* *will not be possible to ensure equal numbers of trial participants who improved v* *ersus* *those who did not* *. N* *or will it be possible to purposively sample trial participants who improved versus those who did not, as the researchers will be blinded to treatment outcome during the sampling phase.”*

1. **The justification for a sample size of 10 therapists.**

To answer our research question for Part B, we judged that we would need to interview a small proportion of participating therapists, not all of them. We estimated that a sample size of ten out of an anticipated 25-30 therapists participating in SURECAN would suffice, provided we include a range of host organisations, core professions, experience and genders in our sampling. We have included this information:

*“* *We aim to recruit 10 therapists participating in the SURECAN trial, out of* *around 25-30 therapists who are participating in the SURECAN trial. This sample size allows us to purposefully sample* *(Campbell et al., 2020)* *, to improve rigour by including a range of views according to differences in host organisations, core professions, levels of experience and genders.”*

1. **Whether the interview guides will be pilot tested prior to recruitment (and if not, why not).**

The interview guides were not pilot-tested prior to recruitment, however, we have added the following clarifying information to the protocol:

*“While the interview guides were not pilot tested, they were developed collaboratively by the study team, drawing on: members’ wide expertise, and interview guides we had developed for the SURECAN pre-pilot study (a small test-run of ACT+, followed by individual interviews with participating patients and ACT+-trained therapists). We also drew from interview guides used in a similar study comparing trial participants’ experiences of improvement and non-improvement for an intervention* *(Cheshire et al., 2020)* *. Additionally, the interview guide for trial participants was reviewed by five of the SURECAN study PPI representatives. Finally, in qualitative research, it should be noted that interview guides are seen as ‘a work in progress’* *(Adams, 2015)* *. It is recommended that guides* *be* *further refined periodically as the researcher conducts interviews in the field,* *as* *emerging insights suggest how* *topics should be refined or added* *.”*

1. **How Patient and Public Involvement members will be involved in the qualitative study.**

The following information has been added to the protocol:

*“Two Patient and Public Involvement (PPI) representatives were grant co-applicants for the SURECAN research programme. Along with other PPI representatives, they have been actively involved in SURECAN through regular programme management meetings as well as specific PPI meetings,* *and through participation in reviewing and commenting on patient-facing materials used in the ACT+ therapy* *sessions.  Five SURECAN PPI representatives reviewed the invitation letter/email, the study information sheet and the interview schedule for trial participants in this qualitative study. Interim findings from this qualitative study will be presented to the SURECAN PPI representatives for debate.”*

1. **Whether specific reporting guidelines will be used when reporting the qualitative study, such as the consolidated criteria for reporting qualitative research.**

While we will be cognisant of the items in the checklist of the consolidated criteria for reporting qualitative research in our reporting, we note recent research that questions the credibility of such checklists (Buus & Perron, 2020). Additionally, their apparently transparent criteria risk undermining the rigour and quality of qualitative research (King, 2021). Thus, we will use the checklist as an advisory only.
